# A Qualitative Study to Explore Perception of Impacts of Preemption of Tobacco Regulation on Counties in Appalachian Tennessee

**DOI:** 10.3390/ijerph17093230

**Published:** 2020-05-06

**Authors:** Hadii M. Mamudu, Fenose Osedeme, Crystal Robertson, Mary Ann Littleton, Daniel Owusu, Liang Wang, Donley T. Studlar

**Affiliations:** 1 Department of Health Services Management and Policy, College of Public Health, East Tennessee State University, P.O Box 70264, Johnson City, TN 37614, USA; 2 Department of Behavioral and Community Health, College of Public Health, East Tennessee State University, P.O Box 70264, Johnson City, TN 37614, USA; osedeme@etsu.edu (F.O.); littleto@etsu.edu (M.A.L.); donleystudlar@gmail.com (D.T.S.); 3 School of Plant Environmental and Soil Sciences, Louisiana State University, Baton Rouge, LA 70808, USA; crystalr501@gmail.com; 4 Tobacco Center of Regulatory Science (GSU TCORS), Georgia State University, Urban Life Building 850, 140 Decatur St, Atlanta, GA 30030, USA; owusud@etsu.edu; 5 Department of Biostatics and Epidemiology, College of Public Health, East Tennessee State University, P.O Box 70659, Johnson City, TN 37614, USA; wangl2@etsu.edu

**Keywords:** preemption, tobacco control, Tennessee, Appalachia, public health

## Abstract

Bottom-up processes, starting at the local government level, are valuable for more-stringent tobacco control measures. The existence of industry-backed state-level tobacco control preemption in states has impeded policy progress within the state and localities/communities. A national public health goal under *Healthy People 2020* is to eliminate state-level preemption across the United States. This study explored individual-level perceptions of the impact of state-level preemption in Appalachian Tennessee—a high-smoking, low-income region. During 2015–2016, a community-engagement project to develop a Population Health Improvement Plan (PHIP) involving over 200 stakeholders and 90 organizations was conducted in Appalachian Tennessee to identify policies/programs to address tobacco use. Using a multifaceted framework approach that focused on prevention, protection, and cessation, interviews and meeting discussions were audio-recorded and transcribed. Content analysis using NVivo 11 was conducted to generate themes. Although the central focus of the PHIP was not preemption, the issue emerged naturally in the discussions as a major concern among participants. Cultural and normative factors in Appalachian Tennessee were identified as key rationales for participants’ aversion to state preemption. Thus, repealing preemption would facilitate culturally tailored and region-specific policies/programs to the high tobacco use among Appalachian Tennessee communities where statewide/nationwide policies/programs have not had the intended impacts.

## 1. Introduction

Tobacco use continues to be the leading preventable cause of morbidity and mortality in the United States (U.S.) [1]. Disparities, however, exist in the prevalence of tobacco use across population subgroups [2,3,4,5] and geographic areas, with the disproportionate burden on people with lower socioeconomic status and rural regions such as Central Appalachia [6,7]. Thus, achieving the *Healthy People 2020* [8] goal of a 12% national adult smoking rate and reducing/eliminating health disparities requires the adoption of population-specific policies; hence, the relevance of examining local tobacco control as it relates to statewide/national policies and programs.

Although the federal government has taken only limited legislative actions, tobacco usage in the U.S. has declined from 42.4% in 1965 to 13.7% in 2018 [8,9,10,11,12]. In a federal system of government, a major reason contributing to this nationwide decline in tobacco use is multilevel governance [13,14,15]. The evidence indicates that states with comprehensive tobacco control programs such as California and Massachusetts have lower tobacco use prevalence than those with fewer policies and less comprehensive programs, such as the Southeastern states, including Tennessee [1,9,16]. However, the constitutional arrangement is such that a higher level of jurisdiction can affect the progress of a policy in a lower jurisdiction by preempting the ability of the lower jurisdiction from advancing policies in the issue area(s) [17].

Evidence indicates that the impact of state preemptive laws on local policies is important because local communities are usually the first and often effective responders to public health concerns resulting from tobacco use [18]. Local policies are usually developed to safeguard the health and wellbeing of the population and, as a result, local tobacco control policies are usually the most innovative and often end up being adopted at the state level [19]. This issue of preemption emerged organically in a Population Health Improvement Plan (PHIP) for tobacco use cessation project involving the eight counties in Appalachian (or Northeast) Tennessee to inform statewide efforts to reduce high prevalence of tobacco use.

This study aimed to explore individual-level perceptions of the impact of state preemption of tobacco regulation on counties in Appalachian Tennessee and the local response to the preemption. The smoking prevalence in Tennessee was 22% (ranked 47th in the country) in 2018 [20], compared to 13.7% nationwide [11], and usage in the predominantly rural counties in Appalachian Tennessee was higher than in the rest of the State, ranging from 21% to 26% [21]. As such, Appalachian Tennessee is part of Truth Initiative-designated “Tobacco Nation” with some of the highest smoking rates in the U.S. [22]. While Tennessee’s preemption has stymied local innovative efforts to address tobacco use, its efforts to address tobacco use have not had the intended impact in Appalachian Tennessee. This is because the culture of resistance to outside policies in Appalachian Tennessee inhibits the adoption or buy-in for such policies [23,24]. This study highlights the perceived effects of preemption as evidence for local and national efforts to reverse tobacco control preemption under the *Healthy People 2020* [25], and illuminates local tobacco control efforts in an environment of preemption. Preemption was enacted in Tennessee in 1994 for three issues—advertising, youth access, and clean indoor air. Advertising refers to restrictions in the display of any tobacco products or samples [26]. Youth access laws limit the sale or distribution of tobacco to young people [26]. Clean indoor air legislation restricts smoking in an environment considered a public space or a workplace [27]. Local governments’ autonomy in enacting smoke-free laws thus have been limited. Despite several attempts to restore local control with policies such as the 2007 Non-smoker Protection Act (NSPA), preemption on local tobacco-control policies is still in existence. All efforts to repeal the state preemption in recent years have failed [28,29,30].

Preemptive tobacco laws in the states arose in the 1980s as a counter strategy from tobacco companies and their allies against the rising state and local attempts at tobacco control. By adopting uniform state standards for tobacco control measures, there could not be “bidding up” with more restrictive standards in some jurisdictions [31,32]. Tobacco control could be contained even if not eliminated, and tobacco industry lobbying could be concentrated at one, central level, the state, rather than the myriad of local jurisdictions whose tobacco control efforts have been encouraged by the federal government through coalition-building [13,31,33].

**Figure A1:**
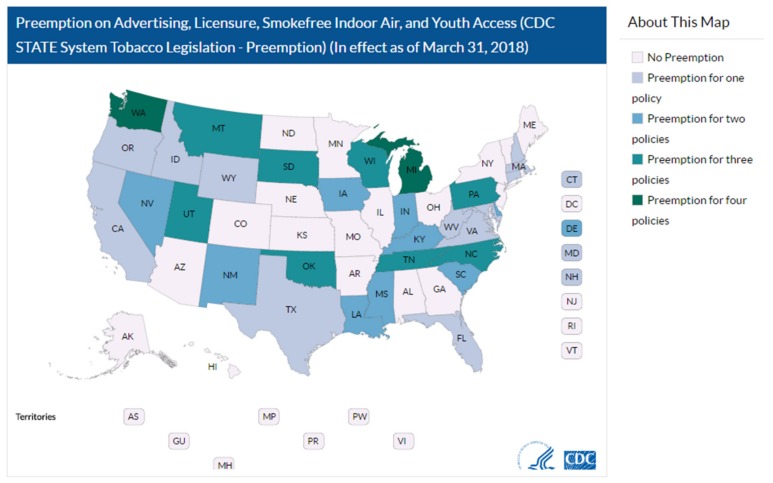
This Geographic Information System (GIS) Map by the U.S. Centers for Disease Control and Prevention (CDC) shows state preemption of four-tobacco control measures: advertising, licensure, smoke-free indoor air, and youth access. Tennessee (TN), where the study was conducted, preempts three of the four tobacco control measures; Advertising, Smoke-free Indoor Air, and Youth Access.

At the peak of this preemptive strategy in the 1990s, over 31 states in the U.S. had some form of preemption in place [25,34,35,36].The mid-1990s saw the battle for tobacco control move to the central government level in the U.S. as the Food and Drug Administration (FDA) began to assert greater control over tobacco regulation. Nevertheless, state preemption did not disappear. Even as of 31 March 2018, 23 of the 50 states had some form of tobacco preemption in place (see Figure A1 in Appendix) with 13 states having explicit preemption, which is when a particular restrictive measure or language is written into legislation [34,37]. Preemption limits flexibility and experimentation in policy, which affects the policy progress in tobacco control in lower levels of political jurisdictions. Therefore, it is a major public health concern [8,38] and key rationale for this study. This has been recognized in successive Healthy People goals for 2000 and 2010 in which one of the goals was elimination of existing preemption provisions [36]. One of the key national public health goals of *Healthy People 2020* is to remove/eliminate preemption of health regulations.

Nonetheless, there is paucity of research on the individual perception and impact of preemption on the policy progress at local jurisdictions [18,39,40,41]. Much of the literature on preemption is more discursive [18,29,42,43] and descriptive [15,44], with few studies that examine the perceived impact of preemption [18,45]. Stark et al. (2007) [45] found, in their study on the impact of clean indoor air law exemptions and preemption policies among nonsmoking bar and restaurant workers, that nonsmoking employees left unprotected from workplace secondhand smoke (SHS) exposure had elevated levels of a tobacco-specific carcinogen in their bodies. Mowery et al. (2012) [18] examined the effect of state provisions preempting local smoking restrictions in enclosed public places and workplaces and found that state preemptive laws were associated with fewer local ordinances restricting smoking, a reduced level of worker protection from SHS, and reduced support for smoke-free policies among both the general public and current smokers. Local smoke-free policies also help to reinforce community norms against tobacco use and smoking [18]. State preemption laws could prevent the shift of social norms that may be generated from the implementation of local smoke-free laws. However, none of these studies analyzes perceptions by individuals of the effects of preemption upon local tobacco control. Thus, this study builds on this sparse literature with focus on Appalachian Tennessee, where no empirical study exists [34], although Tennessee has had explicit preemption on tobacco control regulation since 1994. The state preempts the local control of all publicly owned buildings, although smoke-free laws in private buildings may be enacted by cities and counties, utility districts, airport authorities and special school districts [34].

## 2. Methods

### 2.1. Participants

**Figure A2:**
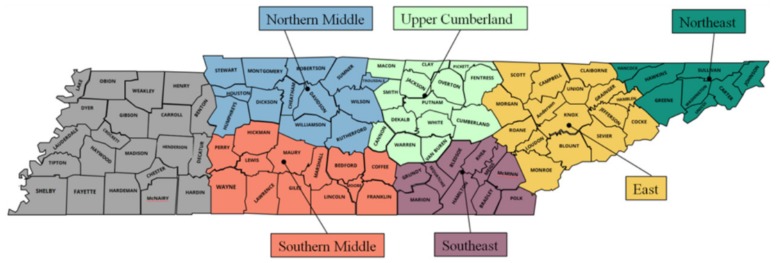
This Map from the Tennessee State Government shows the Counties in Tennessee including the showing the eight northeastern Counties where PHIP project was conducted.

Study participants comprised of a very broad spectrum of stakeholders, including a diverse group of stakeholders in public health programs involved in the PHIP project, 2015–2016. PHIP is a Center of Medicare and Medicaid Services (CMS) project to address tobacco use (including electronic nicotine delivery systems [ENDS] such as e-cigarettes) in Tennessee, with focus on high-prevalence areas of Appalachian Tennessee. Stakeholders for this project included individuals, anti-drug coalitions, representatives of minority populations, representatives of schools and universities, clinics and hospitals, foundations and philanthropies, businesses, the media, special-emphasis institutions such as the health councils, and health departments. Cumulatively, a total of 222 community stakeholders from eight counties Appalachian Tennessee (Carter, Greene, Hancock, Hawkins, and Sullivan, Johnson, Unicoi, and Washington), representing 91 separate organizations participated in the PHIP project (Table 1; Figure A2 in Appendix).

### 2.2. PHIP Process

We utilized a framework of Protection, Prevention, and Cessation (PPC) and a bottom-up, community-based engagement and participatory approach to engage communities in Appalachian Tennessee to assess the problem of tobacco use in the region. Community-based participatory approaches enable communities to be actively involved in addressing complex problems in complex situations [46]. These approaches were used to identify barriers that impede tobacco control and the opportunities that will facilitate tobacco control and delineate local or community efforts to address the burden of tobacco use in the region [24]. While protection focused on population-based policies and programs to protect nonsmokers from exposure to secondhand smoke, prevention focused on preventing youth and young adults from tobacco use initiation, and cessation focused on facilitating tobacco use cessation among tobacco users.

**Figure 1:**
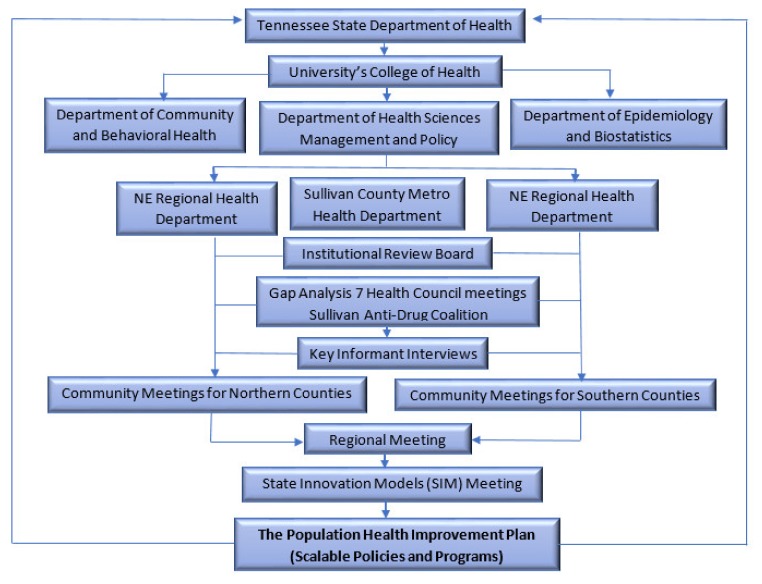
The Population Health Improvement Plan (PHIP) Project Process.

The process started with meetings with local health councils in seven counties (Carter, Greene, Hancock, Hawkins, Johnson, Unicoi, and Washington) and the Sullivan County Anti-Drug Coalition (SCAD; Sullivan County does not have a health council) to identify gaps in local policies and programs to address the high tobacco use (combustible and noncombustible) in the region (see Figure 1).

Subsequently, the snowball method was used to identify stakeholders throughout Appalachian Tennessee for interviews for the PHIP. These stakeholders comprised of individuals within counties who were most involved in tobacco control activities; two key informant interviews were conducted for each of the eight counties. Additional key informant interviews were conducted with regional representatives or members of special populations, including minority groups (n = 20). Additional individuals involved in tobacco control efforts within the county were identified and invited to community meetings.

The data from the health council meetings and the key informant interviews informed two community meetings that were held in the northern part of Appalachian Tennessee, which consists of four counties (Greene, Hancock, Hawkins, and Sullivan) and in the southern part, which consists of the other four counties (Carter, Johnson, Unicoi, and Washington). During these community meetings, secondary data related to tobacco use in the region, information gathered from the health council meetings and key informant interviews related to ongoing efforts, capacity for tobacco control, and opportunities or ways to build capacity were presented to the participants. These community meetings provided an additional chance to gather information within small group discussions or focus groups with representatives from each county. The information obtained from these meetings was compiled and populated within a gap analysis sheet, which was then incorporated into stakeholder findings per County.

At the community meetings, participants were asked to identify key individuals from different community sectors that should be invited to the region-wide stakeholder meeting. Using the snowball recruitment method, these individuals were then contacted and invited to the regional meeting, which was an opportunity to continually engage individuals who were most interested and passionate about tobacco control in the region (see Figure 1).

This PHIP project was approved by the Institutional Review Board (IRB) of East Tennessee State University.

### 2.3. Data Collection and Analysis

An interview/discussion guide was utilized in the data collection process, although priority was given to free flowing of ideas because of the use of bottom-up, community engagement and participatory approach. This guide was informed by the key components of the study’s framework of prevention, protection, and cessation and it entails issues of policies/programs in the communities, integration of tobacco control policies/programs across sectors, challenges and facilitators of tobacco control in communities, and interface between local/community tobacco control and those of the state. Thus, each interview was semi-structured, between 60–90 min long, and employed the same standardized open-ended question guide, probes were used to explore depth and detail. Qualitative data obtained from the key informant interviews and two community meetings were audio-recorded and transcribed by Babel©. Codes guided by evidence from existing research and the Centers for Disease Control and Prevention’s (CDC) *Guide for Comprehensive Tobacco Control Programs* were derived a-priori by one of the co-investigators (MAL). A content analysis of the transcripts based on these codes using both QSR NVivo ver.11 software and manual methods was conducted to generate themes and subthemes. The subthemes were then further condensed by creating overarching themes across counties. The data were summarized in tabular form for each county, and the present study on focused on data related to preemption.

## 3. Results

Analysis of qualitative data from communities’ response to this preemption identified two themes: (1) Cultural and normative factors in Appalachian Tennessee; (2) Concern about the existence of state preemption on tobacco control policies. Both themes had subthemes as high prevalence of tobacco use in Appalachian Tennessee, influence of state preemption on local control, and coping with existing state preemption in Appalachian Tennessee. The meaning of each theme and subtheme is presented with direct quotations from the participants in the following section.

### 3.1. Cultural and Normative Factors in Appalachian Tennessee

Tobacco is ingrained in the culture of Appalachian Tennessee, possibly because the region was a major player in the cultivation and production of tobacco. Participants in the PHIP identified the culture of tobacco use and cultivation as the most salient factor for tobacco use. Representing the overall perspectives of the participants, one person said:

“The cultural history of tobacco in this area. That makes a big difference. When you’re growing and selling tobacco, that was your livelihood, that makes it a lot different then and that’s understandable. That’s cultural, yes” (Stakeholder, Northern County).

Echoing the general sentiment of feeling at the community and regional meetings, a participant said:

“It’s widely accepted. It’s what you do. It’s part of the culture. That smokeless tobacco is out of control here. It’s grandparents to fathers to grandchildren, great-grandchildren. It’s definitely a cyclical thing and that’s what these kids are learning and stick that little piece of dip and let it sit there and park. It’s amazing, especially how young they’re using the smokeless tobacco. That was the number one tobacco product that our fourth and fifth graders were using, or had tried already, starting at age nine, 10 or 11. It’s here. It’s accepted” (Stakeholder, Northern County).

Another participant said,

“Another thing I’ve seen too, culturally, and I know we’re talking about unique populations but, culturally, I have talked to people since I’ve gotten here who were raised on tobacco farms and, as children, their parents would rub tobacco leaves on their skin so they could get nicotine into a child’s body so they could acclimate to touching the tobacco as they worked it, to not get sick. Then by the time they were 10 or 12, the parents encouraged the child to start smoking so they could still handle and work the tobacco. I’ve heard this more than once” (Stakeholder, Northern County).

### 3.2. The Burden of Tobacco Use in Appalachian Tennessee, Compared to Tennessee and the United States

The rate of tobacco use among Appalachian Tennessee residents is higher than that of the State of Tennessee and the entire nation [47,48], which is why the region is part of Truth Initiative-designated “Tobacco Nation” [22]. The participants in the study were generally aware of these disparities in the usage of tobacco products across demographic subgroups and geographic areas. Echoing this overall awareness of the disproportionate burden of tobacco use in Appalachian Tennessee, a stakeholder participant said:

”Smoking [in Northeast or Appalachian Tennessee] is a huge problem in [name of] County” (Stakeholder, Northern County).

Another stakeholder said:

“It’s been a hard issue to tackle [tobacco use] here in [name of] County” (Stakeholder, Southern County).

Highlighting the pervasiveness of tobacco-induced morbidity and mortality in the Appalachian Tennessee region, this person added:

“I’ve never done a presentation at a church that someone wasn’t related to some type of tobacco related illness or affected by that” (Stakeholder, Southern County).

It was identified in this project that tobacco use among people in the region begins as early as five years of age or below. In concurrence with others at the meetings, a participant said:

“I thought that [five years of age] would be a good time to do some of the preventing youth initiation of smoking. It’s grades five through eight. It’s just one school right now” (Stakeholder, Northern County).

Another stakeholder said:

“…but I think for us, it’s seen as people know that kids shouldn’t be smoking, but yet parents will still provide, teachers still allow kids to dip in class. I’m not sure that enforcement is happening in our community like it should” (Stakeholder, Northern County).

### 3.3. Concern about the Existence of State Preemption on Tobacco Control Regulation

A review of participants’ responses during the PHIP study highlights a widespread concern about existing state preemption of tobacco control regulation. In the regional meeting of over 40 individuals representing several stakeholders, including individual citizens, county and regional health departments, health care systems, and educational systems, the move towards the removal of preemption policy was enlisted among the final 25 recommendations to address tobacco use in Tennessee.

During the stakeholders’ interviews, at different times, concerns about the existing state preemption of tobacco control regulation were raised without any prompting. A stakeholder said:

“Preemption is a big thing that needs to be looked at statewide” (Stakeholder, Southern County).

Additionally, strong appeals to the state tobacco preemption in order to facilitate local innovation were highlighted during some interviews with participants. Some responses include:

“NSPA [the Non-Smoker Protection Act] not enough, counties want more control/pre-emption a barrier” (Stakeholder, Southern County).

This person further said:

“Either get rid of the preemption or get [local] exemptions [from the preemption]” (Stakeholder, Northern County).

### 3.4. Influence of State Preemption on Tobacco Control Efforts in Appalachian Tennessee

The impact of the state preemption of local tobacco control efforts was extensively discussed by the participants. In articulating this point, a participant explained:

“…And we have to do that at a state level because of preemption in Tennessee. I think that’s where we need to be stronger to change policies, to change behaviors. An example would be the preemption law in Tennessee. We’re not able to pass any local ordinances in our own community by our residents. It actually has to be a statewide endeavor. I think that we’re challenged” (Stakeholder, Northern County).

Another stakeholder said:

“That is something that the [name of organization] is focused on. We have an implementation plan, and number one on it is to change policy in [name of] County. Where it gets sticky is that the state has a preemption law” (Stakeholder, Northern County).

Reponses from stakeholders highlight the limitations of local ordinances due to preemption. One stakeholder said:

“[Local] governments, I think their hands are tied because of the preemption” (Stakeholder, Southern County).

### 3.5. Coping with State Preemption of Tobacco Control in Appalachian Tennessee

Despite existing restrictions due to the state preemption of tobacco control, Counties in Appalachian Tennessee have continued to find innovative ways for tobacco control policies and programs. During the interviews, a stakeholder revealed that:

“Currently, we’re doing the Knock Tobacco Out of the [public] Park. We actually went to City Council. They didn’t go totally smoke-free, but they’re going to allow us to put the benches in the park that say tobacco-free, or actually in the middle school playground, benches, picnic tables, trash cans, trying to reduce secondhand smoke” (Stakeholder, Southern County).

Another stakeholder mentioned that:

“We developed a TATU group, which was Teens against Tobacco Use. Took them to a training and then they came back and they did some tobacco education at the middle school level for younger students” (Stakeholder, Northern County).

Coalitions and voluntary actions by businesses, health systems, and educational systems continue to identify innovative ways to improve and protect the health of communities while not violating preemption. Through local programs and policies, communities are further engaged in tobacco control activities, thereby advancing social norm change, which is crucial to advance tobacco control locally (Table 2). In this respect, much of the innovative approaches to address the high burden of tobacco use in the region has occurred in the private and voluntary sectors. In particular, local businesses—such as the Local Motor Speedway, Tennessee—have adopted a smoke-free policy that also includes ENDS such as e-cigarettes. Additionally, some local health systems have SFPs to protect the health of its workforce and patients. Their facilities are smoke-free and have no designated areas for smoking. Increasingly, support is growing for local public parks and recreational centers to be smoke-free (Table 2).

## 4. Discussion

Despite national and local efforts to repeal preemption in the U.S. [17,38], 31 states have at least one regulation that preempts tobacco control in local jurisdictions (see Figure A1 in Appendix), with 13 states having explicit preemption [34]. The state of Tennessee is among the states with explicit preemption, and efforts to repeal preemption have failed in legislative committees, specifically within the Agricultural Committee of the State Assembly, regardless of the political party in control of the legislature [28,29,30]. However, over the past decade, the Republican party has controlled both houses of the Tennessee State Assembly and the governorship. Prior studies suggest that preemption has several negative effects, such as the enforcement of tobacco control regulation, civic engagement, and grassroots movements [39,41]—as articulated by a participant who said:

“That [preemption] is something that the [name of organization] is focused on. We have an implementation plan, and number one on it is to change policy in [name of] County. Where it gets sticky is that the state has a preemption law” (Stakeholder, Northern County).

Therefore, this study explored perceptions about the impact of the State of Tennessee preemption on tobacco control in Appalachian Tennessee to illuminate how the policy has affected the progress of tobacco control within localities/communities and inform state/national efforts to repeal/remove preemption.

The negative effects of state tobacco control preemption and disproportionate burden of tobacco use in Appalachian Tennessee emerged organically in grassroots collaborations and local community engagement efforts during the PHIP process. Participants were aware of the state preemption and generally indicated that preemption has negatively affected the progress of tobacco control in the region. As such, there was a strong sentiment at this local level for the repeal of preemption, which supported the position of Mamudu and Veeranki (2013) [29]. However, there were ongoing efforts to deal with the negative efforts of preemption on local tobacco control.

Within local counties, community engagement approaches have proven to be effective in making lasting changes in communities, which is thwarted by the preemption of health regulation. Prior studies have examined how local jurisdictions can deal with the issue of preemption with limited empirical bases [17,41]. Some of these efforts include formations of local coalitions, local governments’ voluntary initiatives and incentivized public programs, and non-governmental organizations’ voluntary health campaigns and programs. However, discussions on the preemption of tobacco control regulation during the PHIP project suggest that these piecemeal and voluntary efforts are not enough to address tobacco use in an environment where usage is highly pervasive such as Appalachian Tennessee, and where there is the need for change in social norms and culture of tobacco use. Evidence from this study indicates that preemption creates constraints for localities about how they can spend federal and state resources to reduce tobacco use, with limited attention to where the resources can make major impacts. These constraints were evident in how the counties in Tennessee, including those in Appalachia, were allowed to spend Master Settlement Agreement money that was disbursed by the state during the PHIP project, although tobacco use prevalence varies across counties and regions of the state [46]. This phenomenon suggests the importance of eliminating preemption of tobacco regulation in Tennessee and elsewhere in the U.S.

Empirical evidence, however, exists about how local jurisdictions are coping with the state of Tennessee preemption of tobacco regulation to address the disproportionately high burden of tobacco use and tobacco-related morbidity and mortality. One of the top issues in Tennessee Department of Health’s State Health Plan is how to address the high prevalence of tobacco use in the State [30]. Regional differences in the prevalence of tobacco use suggest that the efforts of Tennessee Department of Health have disparate and unequal impact in the state [20,49]; therefore, it should consider the elimination of the state preemption as part of the health plan and give the local jurisdictions the authority to protect their own populations from the hazards of tobacco use and secondhand smoke exposure. In this regard, the state role will be creating a framework for tobacco control that sets the floor for tobacco control throughout the state, not the ceiling [29].

Despite the preemption of tobacco regulation in Tennessee, this study identified the resilience of the counties in Appalachian Tennessee to address the disproportionate burden of tobacco use. In particular, the development of evidence-based, culturally tailored programs such as Baby and Me, to reduce the number of pregnant mothers who smoke, has been an innovative approach [24]. This resilience is similar in other regions where preemption of tobacco regulation exists. In Oklahoma, in spite of the near complete preemption in the state [15], community coalitions promoted the adoption of local policies where allowable, with 92 ordinances mirroring state clean indoor air laws and 88 ordinances mirroring state youth access laws. Tobacco-free property policies were adopted by 292 school districts and 309 worksites [30]. In North Carolina, just a 3-month delay in preemption in the early 1990s facilitated the adoption of 89 new regulations [44]. Nevertheless, the evidence from Appalachian Tennessee indicates that these piecemeal local actions amidst preemption of tobacco regulations are not enough to bring about major changes needed to reduce tobacco use, including social norms and culture. The ongoing state struggle between tobacco proponents and control advocates still has consequences.

As an example, the nearby Appalachian state of West Virginia (with only one specific preemption statue on advertising) has allowed community-based local tobacco measures, enacted largely through city councils and county boards of health, to proliferate despite lack of action by the state government. In 2006, there were only four counties out of a total of 55 with 100% nonsmoking regulations for all workplaces, including bars and restaurants; in 2019, this number had grown to 55. This means that over 65 percent of West Virginia’s population is protected by such comprehensive indoor smoking rules, versus zero percent covered in Tennessee. Meanwhile, West Virginia has had to continually rebuff tobacco industry lobbying to extend preemption to youth access [34,50,51].

This study triangulated multiple sources of qualitative data and, as such, certain limitations should be noted in the interpretation of the results. First, the study focused on eight counties in Appalachian Tennessee with the prevalence of tobacco use higher than the state and national average and other unique contextual factors. As such, while the study provides rich contextual information, it is limited by the ability to generalize the findings. Second, the overarching purpose of the PHIP project was to inform the development of tobacco control policies in the state. Therefore, as shown by the stakeholder table (Table 1), participation was skewed towards health-based groups and organizations, limiting the ability to get the perspectives of tobacco companies and affiliated groups. Third, the issue of preemption emerged organically in the PHIP project through the community outreach and engagement. Therefore, while this study demonstrates the impact of preemption on tobacco control at the local/community level and illuminates grassroots frustrations with preemption, it was not specifically designed as a study on state preemption, but rather general tobacco control. Lastly, both the interviews and meeting discussions were conducted in social settings; therefore, the is the possibility of social desirability bias in the presentation of opinions and perspectives about the issues.

Despite these limitations, the study has several strengths, especially involving a critical policy issue (preemption) where there is a paucity of studies involving the impact of the policy on localities/communities and grassroots’ perceptions about it. First, this study focused on elucidating contextual information to inform broader policy change; therefore, it provides rich information that cannot easily be obtained. Second, the issue involved in this study emerged “organically” in the PHIP project to highlight the priorities localities/communities place on a policy that impedes their ability to address issues close to them, the high prevalence of tobacco use and tobacco-induced chronic diseases. Third, the study elicited broad participation through the interviews and the meetings (Table 1), suggesting the priority the localities/communities places on tobacco control, although the region has a history of tobacco production. Fourth, the study triangulated multiple qualitative data, which indicate that the results are well-evidenced and are broadly-based issues of concerns to the localities/communities. Finally, the study confirmed Mowery et al.’s (2012) [18] finding that allowing tobacco control discussions in the localities/communities serves health education and promotion purposes through dialogue and overall community exposure to the issues.

## 5. Conclusions

The evidence from this PHIP project indicates that state preemption needs to be repealed because Appalachians comparatively suffer disproportionately poor health and increased risks of adverse health outcomes. Participants in the project overwhelmingly indicated that the state preemption of tobacco regulation has impeded their ability to develop innovative policies and programs to address the high prevalence of tobacco use and tobacco-induced diseases in Appalachian Tennessee. However, we do not claim that these eight counties are representative of the whole state of Tennessee, or for that matter the Appalachian region, which stretches from Alabama to Maine in the U.S., or even all states with preemption provisions. However, this study is at least suggestive of how local/community residents concerned about tobacco control react to preemption. The evidence from the data indicates the need for region-specific and culturally tailored policies and programs and the manner the issue emerged in the PHIP project demonstrates the deep-seated aversion to the state preemption of tobacco regulation in Appalachian Tennessee. As such, removing state preemption of tobacco regulation could yield the proliferation of more local innovative tobacco control policies to improve the wellbeing of Appalachian residents and the rest of the state [15]. In other words, preemption hinders local tobacco control coalitions, active in Appalachia since the 1980s, from making progress in counties and municipalities, which then might spread to the state level. This bottom-up diffusion has been found to be one of the most effective means of tobacco control [19]; therefore, policy change is terms of repeal/removal of preemption is critically needed with the state playing a role of creating a floor for tobacco control for localities/communities, to avoid any race to the bottom phenomenon. As Cairney et al. (2012) [14] suggest, such policy change will require changes in the policy system in Tennessee such as the shift of tobacco control issues from the Agriculture to the Health Committee in the State Assembly, efforts by health groups at the grassroots level to bubble-up to the state level through outreach to policymakers (especially those in the State Assembly), and favorable opinion for tobacco control at the local levels should be translated into political actions. Indeed, the West Virginia comparison utilized by Studlar [51] shows that even without favorable action on the state level, well-organized and mobilized local groups can have a substantial effect by themselves, but preemption prevents this.

## Figures and Tables

**Table 1:** Stakeholders for PHIP Tobacco Control project in Northeast Tennessee.

Organization/Institution	Title	Gap Analysis	Interviews	Community Meeting	Regional Meeting
Health Councils × 7		✓		✓	✓
Sullivan County Anti-Drug Coalition		✓		✓	✓
Carter County Health Dept.	Health Educator		✓	✓	✓
Carter County Health Dept.	Health Educator		✓		✓
Carter and Johnson County Health Dept.	Director			✓	✓
Carter County	Counselor			✓	
Greene County Health Dept.	Health Educator		✓	✓	✓
Greene County Health Dept.	Director		✓		
Greene County Laughlin Hospital	Wellness Director			✓	
Greene County Schools Coordinated School Health	Coordinator			✓	
Greene County Takoma Hospital	Wellness Director		✓		✓
Greene County—Plus Mark Inc.	Employee Wellness Programs		✓		
Greene County—John Deere Products	Plant Nurse		✓		✓
Hancock Health Dept.	Health Educator		✓	✓	✓
Hancock Coordinated School Health	Coordinator		✓		✓
Hancock County School-based Clinic	Health Educator			✓	
Hawkins County Health Dept.	Director		✓		✓
Hawkins County Health Dept.	Health Educator		✓		✓
Hawkins County—Church Hill Health Dept.	PPI Tobacco Team Leader			✓	
Hawkins County—Church Hill Health Dept.	RDA			✓	✓
Hawkins County Coordinated School Health	Coordinator			✓	
Johnson County Health Dept.	Health Educator		✓	✓	✓
Johnson County ACTION	Executive Director, Prevention Coordinator		✓	✓	✓
Johnson County/Mountain City Nursing Clinic	Site Coordinator			✓	✓
Johnson County—Pregnancy Support Center	Director			✓	✓
Sullivan County Health Dept.	Tobacco Specialist		✓		✓
Sullivan County Health Dept.	Director				✓
Sullivan County Anti-Drug Coalition	Prevention Coordinator		✓	✓	✓
Sullivan County Anti-Drug Coalition	Director			✓	✓
Sullivan County Health Dept.	Tobacco Specialist				✓
Sullivan County	Physician				✓
Sullivan County—Kingsport YMCA	CEO				✓
Unicoi Health Dept.	Health Educator		✓	✓	
Unicoi Health Dept.	Director			✓	✓
Unicoi Hospital (MHSA)	Nurse Manager		✓		✓
Unicoi Project Access	Navigator			✓	✓
Unicoi YMCA	Director			✓	
Washington County Health Dept.	Director		✓	✓	✓
Washington County Health Dept.	Health Educator		✓	✓	✓
American Cancer Society	Community Manager			✓	✓
American Cancer Society	Community Manager			✓	✓
Frontier Health	Wellness Facilitator			✓	✓
Frontier Health	Director		✓		✓
ETSU—Family Medicine	Professor and Head of Clinical Research		✓	✓	✓
ETSU—Nursing Clinics -	Director School-based Clinics		✓	✓	✓
ETSU—Nursing Clinics	Executive Director		✓		
ETSU Wellness Committee	Member				✓
ETSU Human Resources	Director				✓
ETSU Center for Community Outreach	Director				✓
Hispanic Community	ETSU Outreach		✓		✓
Hispanic Community	NE State Spanish Teacher		✓		
Hispanic Community	Tobacco interest				✓
African American Community	ETSU staff		✓		
Mountain States Health Alliance	Regional Outreach Manager				✓
Northeast Regional Health Dept.	Director				✓
Northeast Regional Health Dept.	Health Council Coordinator		✓		✓
Northeast Regional Health Dept	Program Manager		✓		
Niswonger Foundation	Director of Program and Outreach				✓
Wellmont Hospitals	Director Quality				✓
Wellmont Hospitals	Practice Administrator				✓
Wellmont Hospitals	Director of Community				✓
Totals		8	28	66	120

**Table 2:** Local Policies and programs in Central Appalachia during the Era of State Preemption In tobacco control.

S/N	Organization	Description of Policies and Programs
1	**Local Businesses**	
	Crown Laboratories	• Smoke-free campus; must smoke inside cars only, including ENDS. • Optional life insurance policy gives rates based on smoking status through Boston Mutual.
	Bristol Motor Speedway	• Smoke-free policy (including ENDS) that is enforced through the social media, text message.
	Academy Hill Condo Association, Jonesborough	• Smoke-free building policy, requires no smoking by residents in the building
2	**Educational Systems**	
	East Tennessee State University’s Tobacco free policy	• Ban on the use of smoked, smokeless, and any lit product (including ENDS), except in private vehicles. • Campus patrol to ensure compliance. • Noncompliance is progressively penalized. • Standing tobacco committee. • Organization of campus educational events. • Cessation services available and accessible to students. • Continuous research to assess policy efficacy, attitudes, compliance, etc.
	All school systems in the region	• Tobacco free campus, including ENDS
	Teens against Tobacco Use (TATU)	• School based health promotion program, peer-to-peer educational programs for students aged 14–17 years.
	Tar Wars	• School-based 4-h health curriculum targeting youth aged 8–14 years.
	The Michigan Model for Health	• School-based health curriculum implemented by coordinated School Health.
3	**Public Organizations**	
	Johnson City Power Board (JCPB)	• Smoke free campus, including ENDS. • No smoking in JCPB vehicles or in front of customers or out on a job. • Campus has designated smoking areas. • Contracted MSHA nurse that periodically conducts smoking cessation classes and works on a one-on-one basis with employees.
4	**Health Organizations**	
	Mountain States Health Alliance (MSHA)’s nicotine-free workforce and smoke-free campus	• All employees should be nicotine-free. • Nicotine-dependent employees are provided with support for cessation. • Random test for compliance. • Smoke-free hospital campus.
	Woodridge Hospital of Mountain States Health Alliance	• Tobacco-free mental health facility. • No smoking by patients and provides patients with nicotine replacement therapies.
5	**Governmental programs and policies**	
	Smoke-/tobacco-free outdoor public places	• Campaigns to make ball parks and other outdoor places smoke-/tobacco-free.
	Gold Sneaker Initiative	• Developed to augment health and wellness policy in childcare facilities whereby facilities with Gold Sneaker recognition must adopt a tobacco-free policy.
	Clinical Effort Against Second Hand Smoke Exposure (C.E.A.S.E)	• A training module for healthcare providers that seeks to address family tobacco use in a routine and effective manner.
	Unsmokeable	• A movement led by the Sullivan County Regional Health Department to encourage and inspire Sullivan County youth to live a smoke-free life.
	Tennessee Intervention for Pregnant Smokers (TIPS)	• A program that offers cessation assistance to pregnant women and healthcare provider training to enable them to deliver brief smoking cessation counseling and assistance to pregnant patients.
	Power to Quit	• An incentive-based program involving cessation support during pregnancy.
	Freedom from Smoking Cessation	• Classes taught in Washington and Unicoi counties.
	Tobacco Quitline	• A toll-free telephone service offering personalized support for individuals wanting to quit.
6	**Non-Governmental initiatives**	
	Baby and Me Tobacco Free	• An incentive-based program for pregnant women and women with infant children are given vouchers to purchase diapers for staying smoke-free.
	Project Connect	• An adolescent tobacco cessation and reduction program.
